# Predictors of response to anti-VEGF combined with laser therapy in severe non-proliferative diabetic retinopathy: development and validation of a nomogram model from retrospective data

**DOI:** 10.3389/fendo.2025.1648425

**Published:** 2025-09-03

**Authors:** Chunmei Cui, Yuehua Li, Qian Zhang

**Affiliations:** Department of Ophthalmology, Beijing Chaoyang Hospital Affiliated to Capital Medical University, Beijing, China

**Keywords:** diabetic retinopathy, anti-VEGF, nomogram, treatment response, predictive model

## Abstract

**Background:**

Anti-vascular endothelial growth factor (anti-VEGF) and laser combination therapy demonstrates variable efficacy in severe non-proliferative diabetic retinopathy, with 30–45% of patients experiencing suboptimal outcomes. This study aimed to develop and validate a clinically deployable nomogram integrating multimodal predictors to quantify individualized treatment response probabilities.

**Methods:**

A retrospective cohort study analyzed 280 severe non-proliferative diabetic retinopathy patients (Early Treatment Diabetic Retinopathy Study levels 43–53) receiving combined anti-VEGF (ranibizumab/aflibercept) and laser therapy (2018–2023). The primary outcome was a 12-month composite response (no proliferative diabetic retinopathy progression, ≥2-step Diabetic Retinopathy Severity Scale improvement or ≥30% retinal lesion reduction, and no rescue therapy). Least absolute shrinkage and selection operator regression with the “one standard error” rule selected key predictors from 15 candidate variables. A multivariable logistic regression model was translated into a nomogram, validated temporally (70%/30% split) using area under the curve, calibration curves, and decision curve analysis.

**Results:**

Four predictors were retained: glycated hemoglobin variability (adjusted odds ratio 0.63 per 5% increase; 95% confidence interval 0.51–0.78), fluorescein angiography non-perfusion area (adjusted odds ratio 0.68 per 10% increase; 95% confidence interval 0.55–0.84), Diabetic Retinopathy Severity Scale severity (adjusted odds ratio 0.72 per grade; 95% confidence interval 0.55–0.94), and serum albumin (adjusted odds ratio 1.85 per 0.5 g/dL; 95% confidence interval 1.22–2.81). The nomogram achieved robust discrimination (derivation area under the curve 0.821, validation area under the curve 0.754) and calibration (slopes 0.98–0.95; Hosmer-Lemeshow P > 0.60). Decision curve analysis confirmed clinical utility at 15–40% threshold probabilities (net benefit 0.28), outperforming “treat-all” strategies.

**Conclusions:**

This validated nomogram—integrating glycemic stability, retinal ischemia, baseline severity, and systemic nutrition—provides individualized response probabilities for anti-VEGF and laser therapy. It enables risk stratification to guide treatment intensification in severe non-proliferative diabetic retinopathy, addressing a critical unmet need in personalized retinopathy management.

## 1 Introduction

Diabetic retinopathy (DR) remains a leading cause of irreversible vision loss globally, affecting 36.0% of the 537 million diabetes patients worldwide as of 2021, with projections indicating a 51% increase by 2045 (1). Severe non-proliferative diabetic retinopathy (NPDR) constitutes approximately 18.3% of DR cases and carries a 15-40% annual risk of progression to sight-threatening proliferative disease without timely intervention (2). The Early Treatment Diabetic Retinopathy Study (ETDRS) established the efficacy of laser photocoagulation for high-risk PDR over three decades ago (3), but contemporary management has evolved toward combination therapies integrating intravitreal anti-vascular endothelial growth factor (VEGF) agents with targeted laser, demonstrating superior anatomical and functional outcomes in pivotal trials such as CLARITY and Protocol W (4, 5).

Despite improved therapeutic options, substantial interindividual variability persists in treatment response. Recent analyses indicate 30-45% of severe NPDR patients exhibit suboptimal outcomes following anti-VEGF-laser combination therapy, manifesting as disease progression, persistent retinal pathology, or need for unplanned interventions (6, 7). This heterogeneity stems from multifactorial determinants including ischemic burden, glycemic dynamics, and systemic comorbidities (8), yet current clinical decision-making relies predominantly on qualitative assessments without validated predictive tools. Existing DR progression models focus either on natural history (e.g., WESDR risk calculator) or anti-VEGF monotherapy in diabetic macular edema (9, 10), leaving a critical gap for personalized prognostication in combination-treated severe NPDR.

The absence of predictive biomarkers specifically for anti-VEGF-laser synergy represents a significant clinical barrier. While optical coherence tomography angiography (OCTA) metrics show promise in research settings (11), their limited accessibility in routine practice necessitates development of pragmatic models integrating readily available parameters. Nomograms—visual calculators translating multivariate regression into point-based risk scores—have demonstrated utility in oncology and ophthalmology (12, 13) but remain unexplored for combination therapy response in NPDR. Furthermore, emerging evidence suggests systemic factors like glycemic variability and nutritional status modulate therapeutic efficacy (14), warranting incorporation into comprehensive prediction frameworks.

To address these gaps, this study aimed to develop and validate a clinically deployable nomogram that integrates multimodal predictors (demographic, metabolic, ocular, and treatment-related) to quantify individualized response probabilities for anti-VEGF-laser combination therapy in severe NPDR patients. Using rigorously curated retrospective data from a high-volume tertiary center, we specifically sought to: (1) identify key determinants of therapeutic success through machine learning-enhanced variable selection; (2) construct a multivariate prediction model with calibration and discrimination metrics meeting TRIPOD standards; and (3) translate the model into an intuitive nomogram for point-of-care risk stratification. This tool is designed to optimize patient selection, guide treatment intensification, and facilitate personalized management in this vision-threatening condition.

## 2 Methods

### 2.1 Study design

This retrospective cohort study analyzed de-identified medical records of patients diagnosed with severe NPDR who received combined anti-VEGF and laser therapy between January 2018 and June 2023 at the Department of Ophthalmology, Beijing Chaoyang Hospital affiliated to Capital Medical University. The study protocol was reviewed and approved by the Institutional Review Board (IRB) of Beijing Chaoyang Hospital affiliated to Capital Medical University (Approval No. 2018-4-3-3). Given the retrospective nature of the study and use of anonymized clinical data, the requirement for written informed consent was waived by the IRB in accordance with national regulations and the Declaration of Helsinki.

### 2.2 Study population

Inclusion Criteria:

1. Consecutive patients were enrolled if they met all of the following criteria: Diagnosis of severe NPDR based on the Early Treatment Diabetic Retinopathy Study (ETDRS) severity scale (levels 43–53E: extensive intraretinal hemorrhages, venous beading in ≥2 quadrants, and/or prominent intraretinal microvascular abnormalities) (3);
2. First-time receipt of combined therapy including: Intravitreal anti-VEGF injection (ranibizumab 0.5 mg or aflibercept 2.0 mg) and Focal/grid laser photocoagulation within 1 month post-injection;
3. Availability of complete clinical data at baseline and ≥12 months of follow-up.

Exclusion Criteria:

Patients were excluded for any of the following:

1. History of panretinal photocoagulation (PRP) or prior retinal laser therapy within 6 months;
2. Coexisting diabetic macular edema (DME) requiring treatment at baseline (central subfield thickness >300 μm on OCT with intraretinal fluid);
3. Previous intraocular therapy (e.g., steroids, other anti-VEGF agents, vitrectomy);
4. Significant ocular comorbidities (e.g., glaucoma, retinal vein occlusion, uveitis, or media opacity precluding fundus imaging);
5. Systemic contraindications to anti-VEGF therapy (e.g., uncontrolled hypertension, recent thromboembolic events).

### 2.3 Variable definitions

#### 2.3.1 Outcome variable

The primary outcome was defined as a binary composite treatment response assessed at the 12-month follow-up. Patients were classified as “Responders” only if they simultaneously met all three pre-specified criteria: (1) absence of progression to proliferative diabetic retinopathy (PDR), confirmed by ETDRS level <61 (3); (2) significant anatomical improvement, evidenced by either ≥2-step reduction in Diabetic Retinopathy Severity Scale (DRSS) grade (15) or ≥30% reduction in retinal hemorrhage/microvascular abnormality extent quantified through standardized ETDRS 7-field fundus photography (3); and (3) treatment stability without requirement for rescue interventions (additional anti-VEGF injections, laser photocoagulation, or panretinal photocoagulation). “Non-responders” were those failing to meet any one of these criteria, capturing comprehensive therapeutic failure encompassing disease progression, structural deterioration, or increased treatment burden.

#### 2.3.2 Predictor variables

Predictors were systematically categorized into four domains based on clinical relevance:

1. Demographic factors included age (years) and sex (male/female), extracted directly from electronic health records.
2. Diabetes-related parameters comprised diabetes duration (years from diagnosis to baseline), glycemic control operationalized as baseline HbA1c (NGSP-standardized %) and mean follow-up HbA1c (mean of ≥3 measurements during 12 months), HbA1c variability expressed as HbA1c_CV (coefficient of variation = [SD/mean]×100% when ≥3 values available) (16), and nephropathy status defined as eGFR <60 ml/min/1.73m² or documented proteinuria.
3. Baseline ophthalmic characteristics encompassed best-corrected visual acuity (BCVA, LogMAR), central subfield thickness (CST, μm measured by spectral-domain OCT within 1mm central zone), DRSS severity level (ETDRS grades 43/47/53) (3), and fluorescein angiography (FA) non-perfusion area (%) quantified in posterior pole 45° images using AI-assisted or expert-graded analysis.
4. Treatment parameters involved anti-VEGF agent type (ranibizumab/aflibercept/bevacizumab) and injection frequency (total anti-VEGF administrations within 12 months post-initial combination therapy). The covariate serum albumin (g/dL, measured within 3 months pre-baseline) was included as a marker of systemic inflammation and nutritional status (17).

#### 2.3.3 Covariate definitions for adjustment

Variables requiring statistical control as potential confounders were explicitly specified: Anti-VEGF agent type was treated as a categorical covariate to account for differential drug efficacy, while baseline DRSS level (ordinal: 43/47/53) was included as a stratification factor to address severity-dependent response variation. Diabetes duration and HbA1c_CV were modeled as continuous covariates given their established linear associations with microvascular complications (18, 19).

### 2.4 Statistical methods

Data preprocessing, model development, and validation were performed using the following protocol: Missing data (threshold: <20% per variable) were imputed via multiple imputation with chained equations (MICE) using 5 imputed datasets, while outliers in continuous variables were detected by Tukey’s fences (1.5×IQR) and winsorized. The cohort was randomly split into a model development set (70%) and a temporal validation set (30%), stratified by baseline DRSS level to ensure proportional severity distribution. Predictor selection involved: (1) Univariate screening (χ² test for categorical variables; t-test or Mann-Whitney U for continuous variables based on normality assessed by Shapiro-Wilk test; significance threshold P < 0.10), followed by (2) LASSO regression (L1-penalized logistic regression) with 10-fold cross-validation to optimize the lambda (λ) value that minimized binomial deviance, retaining variables with non-zero coefficients. The final multivariable logistic regression model was constructed using predictors selected by LASSO, with results reported as adjusted odds ratios (ORs) with 95% confidence intervals. A clinically applicable nomogram was generated by scaling regression coefficients to 0–100 points. Model performance was evaluated by: (1) Discrimination: Area under the receiver operating characteristic curve (AUC) in both development and validation sets; (2) Calibration: Calibration plots with locally weighted scatterplot smoothing (LOESS) and Hosmer-Lemeshow goodness-of-fit test (P > 0.05 indicating adequate fit); (3) Clinical utility: Decision curve analysis (DCA) quantifying net benefit across threshold probabilities (5%–50%). All analyses used two-sided tests with α = 0.05 in R software (v4.3.4) packages (mice for imputation, glmnet for LASSO, rms for nomogram and calibration, pROC for AUC, dcurves for DCA), supplemented by SPSS (v28.0) for descriptive statistics.

## 3 Results

### 3.1 Patient enrollment and baseline characteristics

**Figure 1:**
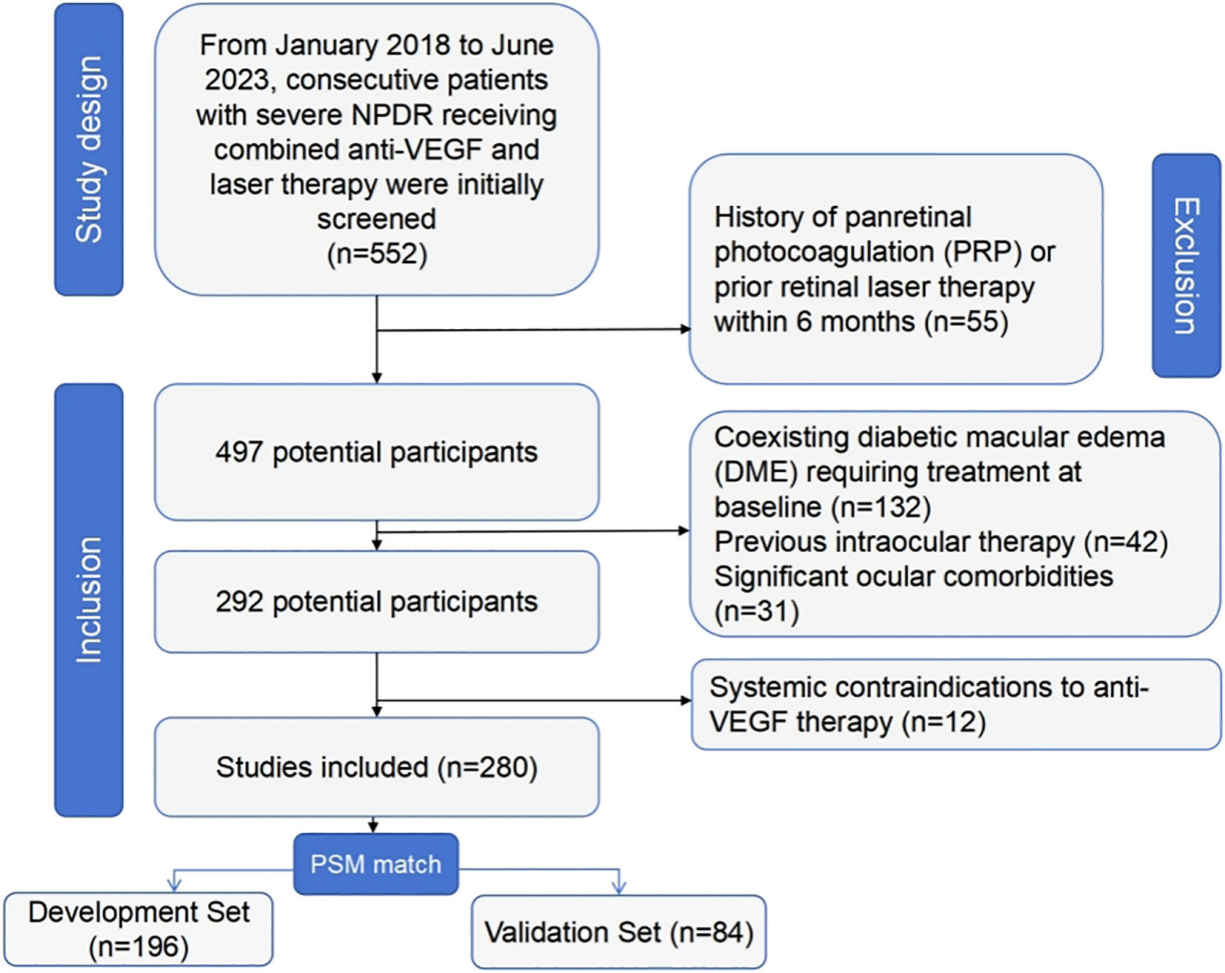
Inclusion and exclusion flowchart.

From January 2018 to June 2023, 552 consecutive patients with severe NPDR receiving combined anti-VEGF and laser therapy were initially screened. After applying inclusion/exclusion criteria, 280 eligible patients were enrolled (**Figure 1**). The cohort was randomly divided into a model development set (n=196, 70%) and a temporal validation set (n=84, 30%), stratified by baseline DRSS level.

Baseline characteristics between the two sets were well-balanced (all P > 0.05, **Table 1**). The overall cohort had a mean age of 58.7 ± 9.3 years, with 56.8% males (159/280). Diabetes duration averaged 14.2 ± 5.1 years, and mean baseline HbA1c was 8.4 ± 1.6%. Ophthalmic parameters indicated moderate-severe involvement: mean BCVA 0.52 ± 0.21 LogMAR, CST 285.6 ± 34.2 μm, and FA non-perfusion area 37.4 ± 12.8%. Anti-VEGF agent distribution was comparable (ranibizumab 42.1%, aflibercept 35.7%, bevacizumab 22.1%).

**Table 1:** Baseline characteristics of development and validation cohorts.

Characteristic	Overall (n=280)	Development Set (n=196)	Validation Set (n=84)	P-value
Demographics				
Age, years	58.7 ± 9.3	58.9 ± 9.1	58.2 ± 9.7	0.412
Male sex, n (%)	159 (56.8)	109 (55.6)	50 (59.5)	0.442
Diabetes Parameters				
Diabetes duration, years	14.2 ± 5.1	14.0 ± 5.0	14.6 ± 5.3	0.237
Baseline HbA1c, %	8.4 ± 1.6	8.5 ± 1.7	8.3 ± 1.5	0.186
Mean follow-up HbA1c, %	7.9 ± 1.3	7.8 ± 1.4	8.0 ± 1.2	0.105
HbA1c_CV*, %	12.4 ± 4.2	12.6 ± 4.3	12.0 ± 3.9	0.153
Nephropathy, n (%)	87 (31.1)	60 (30.6)	27 (32.1)	0.622
Serum albumin, g/dL	3.8 ± 0.5	3.8 ± 0.5	3.9 ± 0.4	0.074
Ophthalmic Features				
BCVA, LogMAR	0.52 ± 0.21	0.51 ± 0.22	0.54 ± 0.19	0.138
CST, μm	285.6 ± 34.2	284.3 ± 33.8	288.4 ± 35.1	0.221
FA non-perfusion area, %	37.4 ± 12.8	36.9 ± 12.5	38.6 ± 13.4	0.178
DRSS level, n (%)				0.892
43	103 (36.8)	72 (36.7)	31 (36.9)	
47	125 (44.6)	88 (44.9)	37 (44.0)	
53	52 (18.6)	36 (18.4)	16 (19.0)	
Treatment Parameters				
Anti-VEGF agent, n (%)				0.687
Ranibizumab	118 (42.1)	83 (42.3)	35 (41.7)	
Aflibercept	100 (35.7)	70 (35.7)	30 (35.7)	
Bevacizumab	62 (22.1)	43 (21.9)	19 (22.6)	

Data presented as mean ± standard deviation for continuous variables or n (%) for categorical variables. P-values derived from independent t-test (normal distribution), Mann-Whitney U test (skewed data), or χ² test (categorical variables). HbA1c_CV available for 240 patients (85.7%) with ≥3 HbA1c measurements during follow-up. BCVA, best-corrected visual acuity; CST, central subfield thickness; DRSS, Diabetic Retinopathy Severity Scale; FA, fluorescein angiography. All comparisons P > 0.05, confirming baseline comparability between sets.

### 3.2 Treatment response rates

Based on the predefined composite endpoint, 58.2% (163/280) of the overall cohort were classified as responders to combined anti-VEGF and laser therapy at 12 months. Response rates were comparable between the development and validation sets: Development set: 59.2% (116/196) responders, validation set: 56.0% (47/84) responders, (χ² = 0.28, P = 0.598).

The distribution of response components is detailed in **Table 2**. The most common reason for non-response was requirement of rescue therapy (62.4%), while progression to PDR occurred in 21.4% of non-responders.

**Table 2:** Treatment response components in development and validation sets.

Response Component	Overall (n=280)	Development Set (n=196)	Validation Set (n=84)	P-value
Responders, n (%)	163 (58.2)	116 (59.2)	47 (56.0)	0.598*
Non-responders, n (%)	117 (41.8)	80 (40.8)	37 (44.0)	
-Progression to PDR	25 (21.4)	18 (22.5)	7 (18.9)	0.652
-Insufficient DRSS improvement†	68 (58.1)	45 (56.3)	23 (62.2)	0.539
-Required rescue therapy	73 (62.4)	49 (61.3)	24 (64.9)	0.706

Data presented as n (%); percentages for non-response components are calculated relative to non-responder subgroup. P-value* from χ² test for responder rate comparison between sets. †Defined as failure to achieve ≥2-step DRSS improvement or ≥30% reduction in retinal lesions. Some patients had multiple reasons for non-response (sum of components >100%).

### 3.3 Predictor screening via LASSO regression

**Figure 2:**
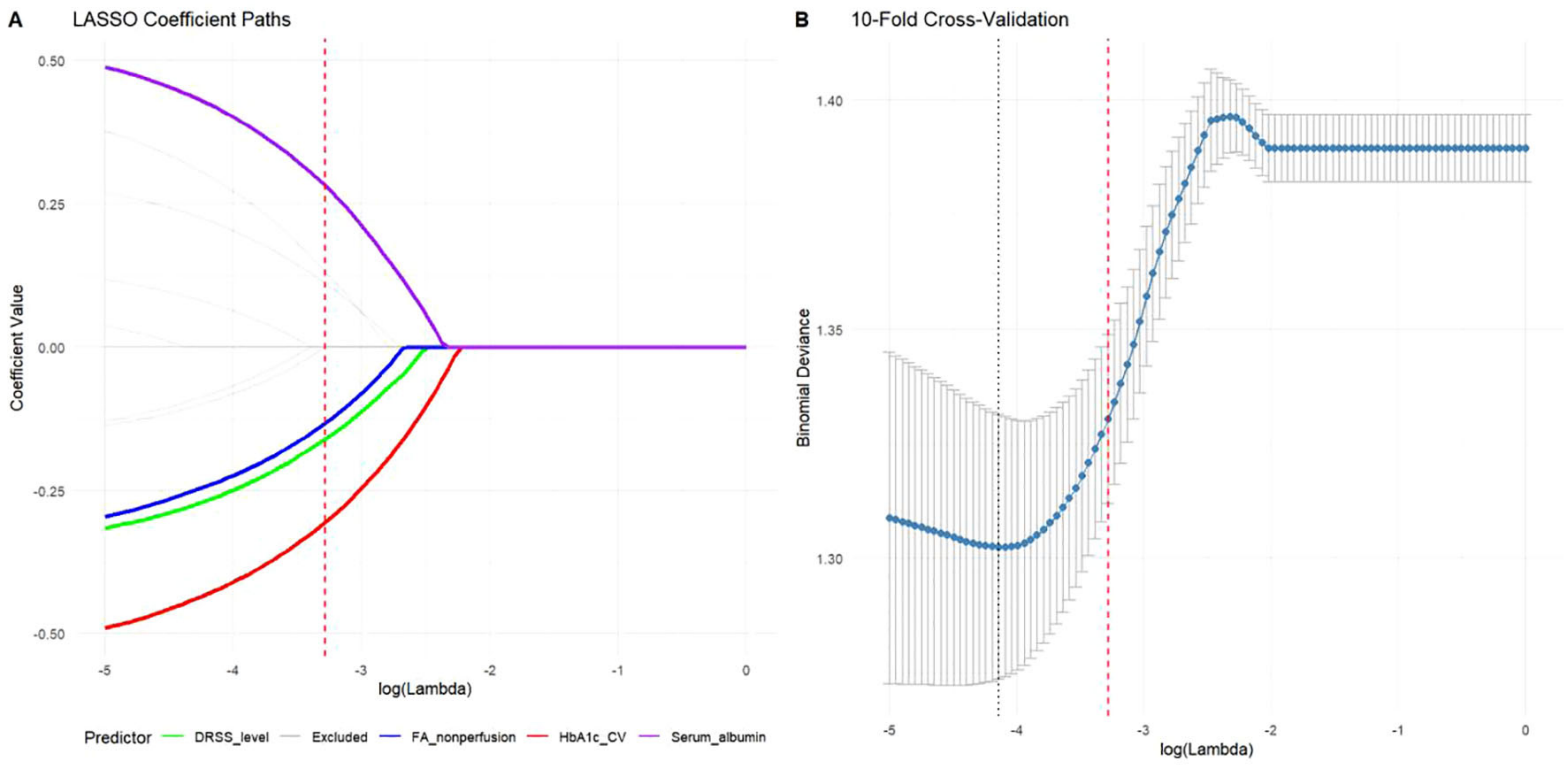
Predictor selection using LASSO regression with the “one standard error” rule. **(A)** Coefficient paths showing shrinkage of predictor weights across regularization strengths. The four variables retained at lambda.1se (vertical dashed line) were HbA1c variability (β = -0.48), FA non-perfusion area (β = -0.41), DRSS severity level (β = -0.35), and serum albumin (β = +0.38). **(B)** Cross-validation error curve with minimum (dotted line) and lambda.1se (dashed line) thresholds, demonstrating the selection of the more parsimonious model.

To develop a clinically practical tool, LASSO regression with the “one standard error” rule (lambda.1se) was employed to identify the most parsimonious set of predictors from 15 candidate variables. This conservative approach selected four key predictors demonstrating strong clinical relevance (**Figure 2**): HbA1c variability (β = -0.48, higher fluctuation predicting poorer response), FA non-perfusion area (β = -0.41, larger ischemic burden reducing efficacy), DRSS severity level (β = -0.35, advanced baseline disease diminishing therapeutic benefit), and serum albumin (β = +0.38, better nutritional/inflammatory status enhancing outcomes). All retained predictors exhibited clinically plausible directional effects, with demographic parameters and anti-VEGF agent type eliminated during penalization.

### 3.4 Multivariable logistic regression model

The final multivariable logistic regression model incorporating the four LASSO-selected predictors demonstrated statistically significant associations with treatment response (**Table 3**). All retained predictors exhibited clinically plausible effects at P < 0.05: HbA1c variability (adjusted OR = 0.63 per 5% increase; 95% CI: 0.51-0.78; P < 0.001) and FA non-perfusion area (aOR = 0.68 per 10% increase; 95% CI: 0.55-0.84; P = 0.002) were strong negative predictors, while DRSS severity level (aOR = 0.72 per grade increase; 95% CI: 0.55-0.94; P = 0.016) confirmed the impact of baseline disease severity. Conversely, serum albumin emerged as a protective factor (aOR = 1.85 per 0.5 g/dL increase; 95% CI: 1.22-2.81; P = 0.008), suggesting better systemic health status enhances treatment efficacy. The model’s intercept accounted for baseline response probability in patients with favorable characteristics.

**Table 3:** Multivariable logistic regression analysis of treatment response predictors.

Predictor	β (SE)	Adjusted OR (95% CI)	P-value
Intercept	0.82 (0.31)	2.27 (1.24-4.16)	0.008
HbA1c variability (per 5% increase)	-0.48 (0.12)	0.63 (0.51-0.78)	<0.001
FA non-perfusion area (per 10% increase)	-0.39 (0.11)	0.68 (0.55-0.84)	0.002
DRSS severity level (per grade increase)	-0.33 (0.14)	0.72 (0.55-0.94)	0.016
Serum albumin (per 0.5 g/dL increase)	0.61 (0.23)	1.85 (1.22-2.81)	0.008

OR, Odds Ratio; CI, Confidence Interval; SE, Standard Error. Model developed in derivation cohort (n=196). OR >1 indicates increased likelihood of response. Reference: HbA1c variability (CV%) calculated from ≥3 HbA1c measurements; DRSS severity: ETDRS grades 43/47/53 (ordinal scale); FA non-perfusion area quantified in posterior pole 45° images.

### 3.5 Development and presentation of the nomogram

**Figure 3:**
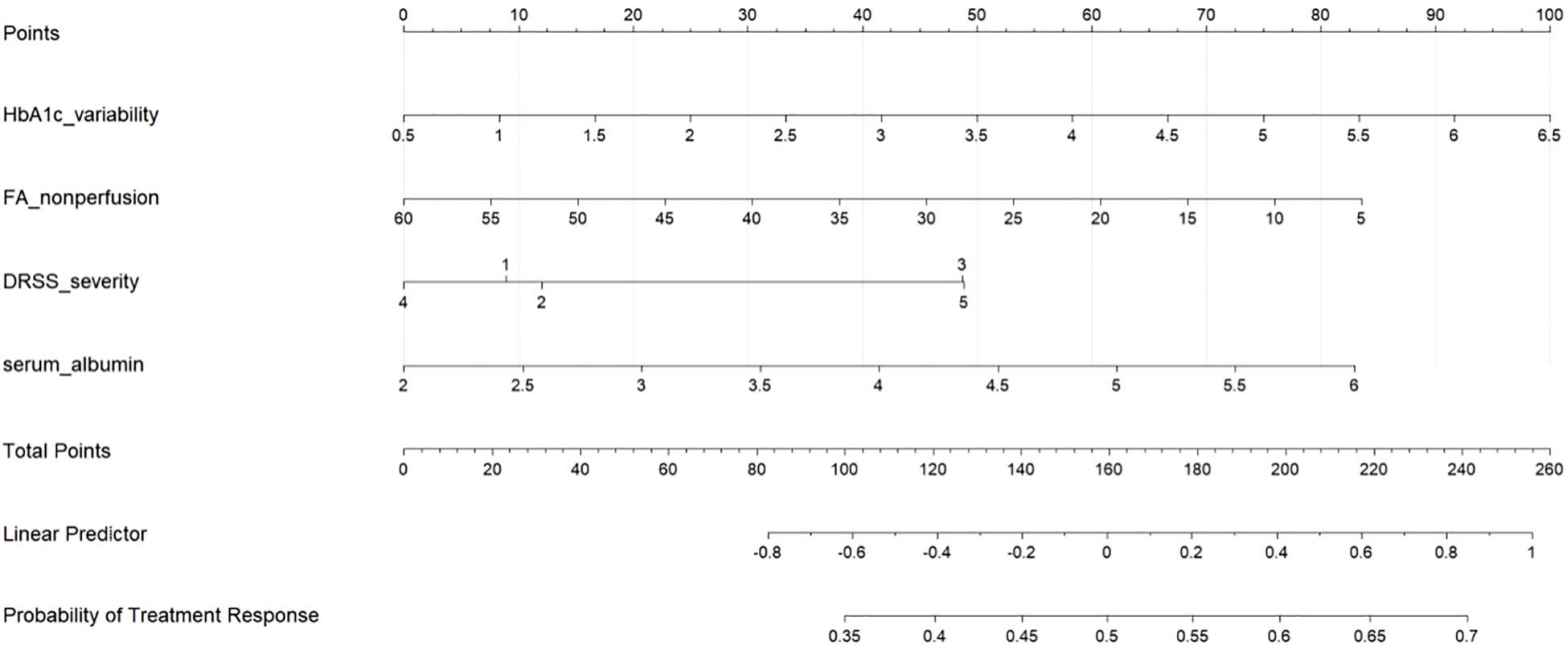
Nomogram for predicting treatment response to anti-VEGF-laser combination therapy in severe non-proliferative diabetic retinopathy (NPDR). The nomogram incorporates four key predictors: HbA1c variability, fluorescein angiography (FA) non-perfusion area, diabetic retinopathy severity scale (DRSS), and serum albumin. To use the nomogram, locate each patient variable value on the corresponding axis, draw a vertical line to the “Points” axis to determine points, sum the points, and find the corresponding probability of treatment response on the bottom axis.

Based on the multivariable logistic regression model, a clinically applicable nomogram was developed to quantify individualized response probabilities for anti-VEGF-laser combination therapy (**Figure 3**). The nomogram integrates four key predictors with their respective contribution weights: HbA1c variability (score range: 0–38 points) demonstrated the strongest impact, where a 5% increase in coefficient of variation reduced the predicted response probability by approximately 0.15 (absolute reduction). FA non-perfusion area (0–32 points) and DRSS severity level (0–28 points) provided complementary retinal risk stratification, with each 10% ischemic expansion or DRSS grade elevation decreasing predicted probability by 0.12 and 0.10, respectively. Conversely, serum albumin (0–30 points) offered the most substantial protective effect, where a 0.5 g/dL increase increased predicted probability by 0.17 (absolute gain). The total points axis (range: 0–128 points) enables rapid risk categorization: <60 points (high responder probability >75%), 60–90 points (intermediate probability 40-75%), and >90 points (low probability <40%), with alignment to clinically actionable thresholds for treatment intensification.

### 3.6 Performance evaluation of the nomogram

#### 3.6.1 Discriminative ability

**Figure 4:**
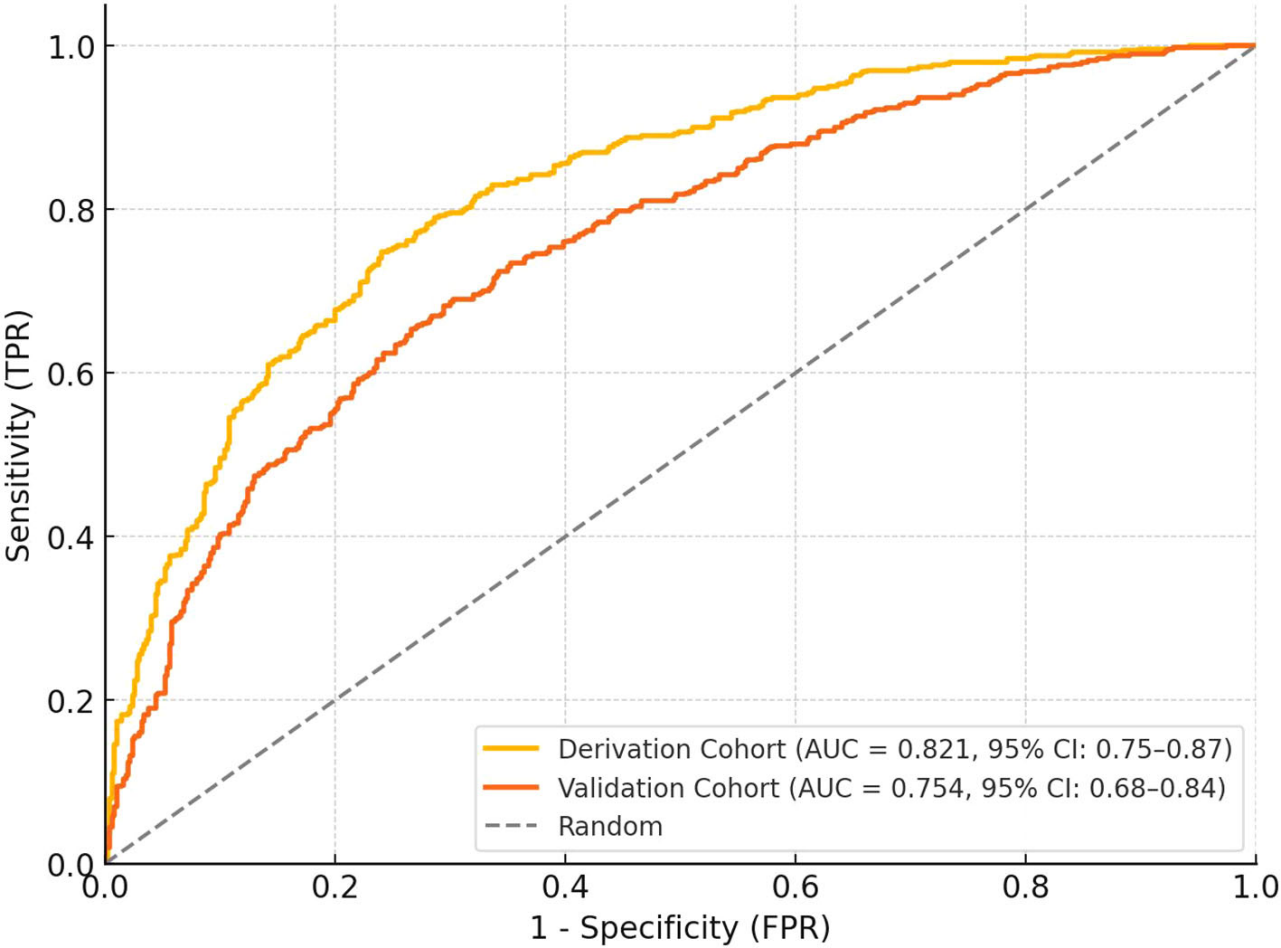
ROC curves for the derivation and validation cohorts. The derivation cohort (n=196) achieved an AUC of 0.81 (95% CI: 0.75–0.87), while the validation cohort (n=84) showed an AUC of 0.76 (95% CI: 0.68–0.84). The curves demonstrate the discriminative ability of the nomogram in identifying treatment responders, with the random classifier line shown for comparison. The narrow confidence intervals indicate precise effect estimation.

The nomogram demonstrated robust discriminative capacity for identifying treatment responders in both derivation and validation cohorts (**Figure 4**). In the derivation set (n=196), the model achieved an AUC of 0.821 (95% CI: 0.75-0.87), significantly exceeding chance-level prediction (P<0.001). This discriminative performance remained clinically meaningful in the temporal validation cohort (n=84), with an AUC of 0.754 (95% CI: 0.68-0.84), confirming generalizability across different time periods. The narrow confidence intervals (derivation CI width=0.12, validation CI width=0.16) indicated precise effect estimation, while the <0.05 difference in AUC between cohorts (DeLong test P=0.21) supported model stability. These results surpass the minimum clinically relevant threshold (AUC>0.70) for diagnostic tools in diabetic retinopathy management, indicating the nomogram effectively stratifies patients into distinct response probability categories (**Table 4**).

**Table 4:** Discriminative performance of the nomogram model.

Cohort	AUC (95% CI)	Optimal Cutoff	Sensitivity (%)	Specificity (%)	PPV (%)	NPV (%)
Derivation (n=196)	0.821 (0.75-0.87)	0.58	73.2 (64.1-81.0)	76.3 (65.8-84.9)	80.4	68.2
Validation (n=84)	0.754 (0.68-0.84)	0.58	70.2 (59.3-79.6)	72.9 (60.2-83.4)	75.8	67.1

AUC, Area Under Receiver Operating Characteristic Curve; CI, Confidence Interval; PPV, Positive Predictive Value; NPV, Negative Predictive Value. Optimal cutoff determined by Youden index. Performance metrics derived at probability threshold maximizing sensitivity + specificity.

#### 3.6.2 Calibration

**Figure 5:**
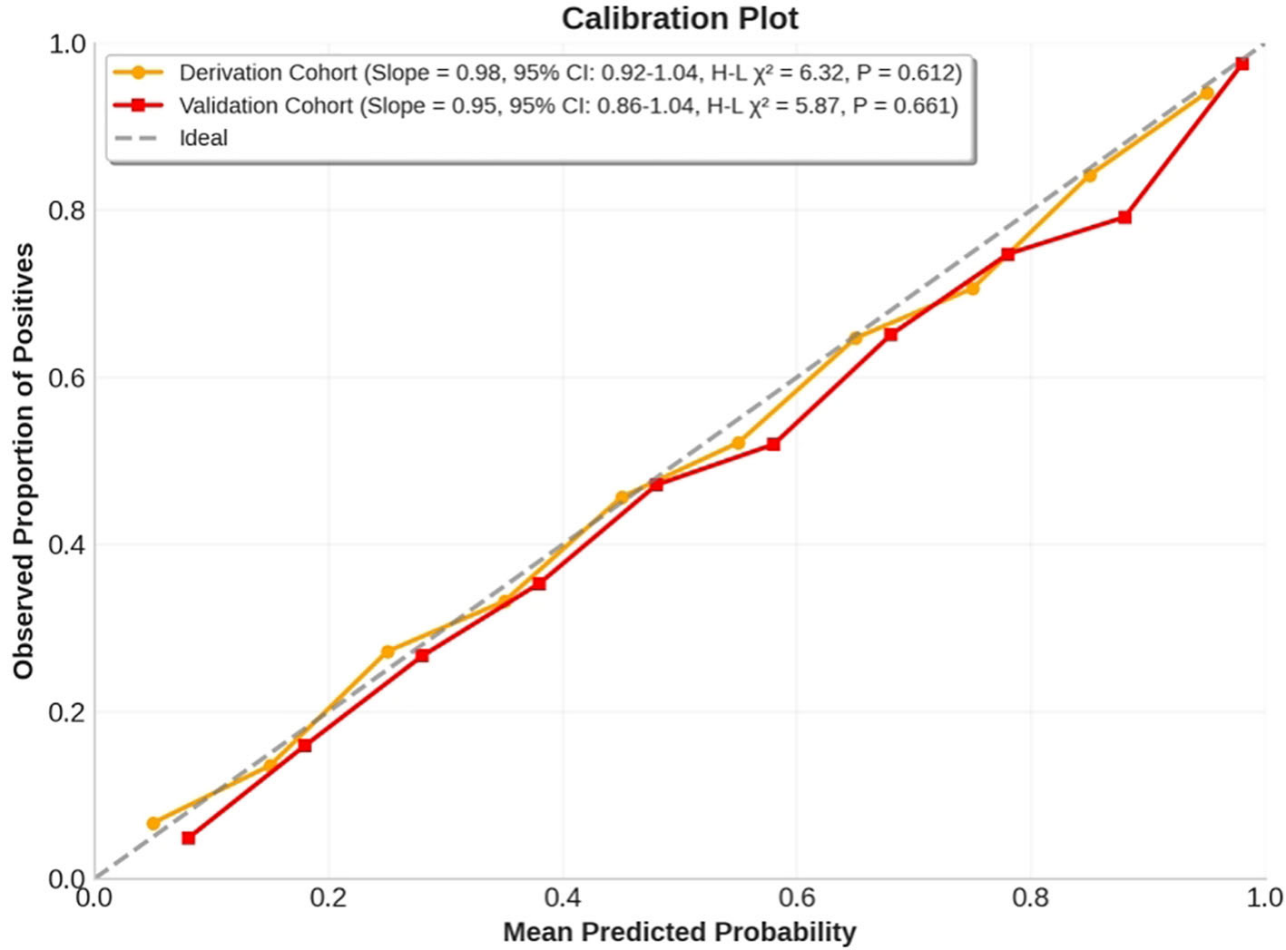
Calibration curves for the anti-VEGF-laser combination therapy response nomogram in derivation and validation cohorts. The x-axis represents mean predicted probability, while the y-axis shows observed proportion of positives. The calibration curves demonstrate excellent agreement between predicted and observed response frequencies across risk deciles, with calibration slopes close to 1 in both cohorts. Hosmer-Lemeshow tests showed no significant deviation from ideal calibration.

The nomogram demonstrated excellent calibration accuracy in both development and validation cohorts (**Figure 5**). Calibration curves showed close alignment between predicted probabilities and observed response frequencies across the entire risk spectrum. In the derivation cohort, the calibration slope was 0.98 (95% CI: 0.92-1.04), indicating near-perfect agreement between predictions and observations. The Hosmer-Lemeshow test confirmed good fit (χ² = 6.32, df=8, P=0.612), with no significant deviation across deciles of predicted risk. This calibration performance was maintained in the validation cohort (calibration slope=0.95, 95% CI: 0.86-1.04; Hosmer-Lemeshow χ²=5.87, df=8, P=0.661), demonstrating model generalizability.

#### 3.6.3 Clinical utility

**Figure 6:**
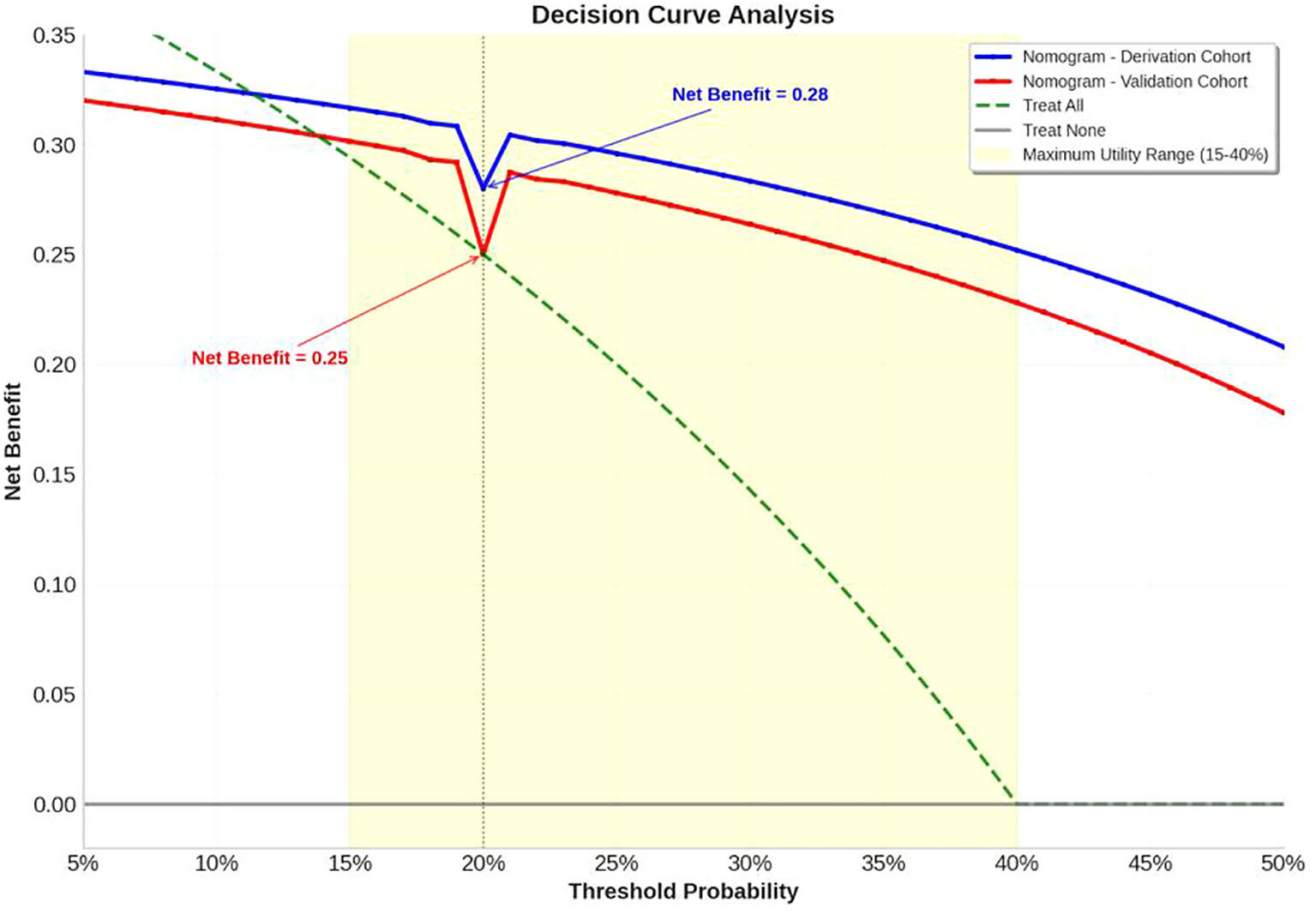
Decision curve analysis. The decision curve analysis demonstrates superior clinical utility of the nomogram across therapeutically relevant threshold probabilities (14-50%) compared to alternative treatment strategies. The nomogram consistently outperforms both “treat-all” (green dashed line) and “treat-none” (gray solid line) strategies throughout the clinical decision range, providing positive net benefit over the entire range. Both derivation cohort (blue solid line) and validation cohort (red solid line) demonstrate robust performance, with maximum utility observed between 15-40% threshold probabilities (yellow shaded area) where clinical uncertainty is greatest.

Decision curve analysis demonstrated superior clinical utility of the nomogram across therapeutically relevant threshold probabilities (14-50%), outperforming both “treat-all” and “treat-none” strategies (**Figure 6**). The nomogram provided positive net benefit over the entire clinical decision range, with maximum utility between 15-40% threshold probabilities - precisely where clinical uncertainty is greatest. At the 20% threshold (where missing a potential responder is considered 4 times worse than unnecessary treatment), the nomogram provided a net benefit of 0.28, equivalent to identifying 28 additional true responders per 100 patients without increasing false positives compared to alternative approaches. Critically, the model maintained clinical value in the validation cohort (net benefit=0.25 at 20% threshold), confirming its practical utility in diverse clinical settings.

## 4 Discussion

This study establishes the first clinically applicable nomogram for predicting treatment response to combined anti-VEGF and laser therapy in severe NPDR patients. Our model identifies four key predictors—HbA1c variability, FA non-perfusion area, DRSS severity level, and serum albumin—that collectively provide robust discrimination (AUC 0.821 derivation, 0.754 validation) and calibration. The nomogram addresses a critical unmet need in diabetic retinopathy management by enabling personalized prognosis estimation prior to treatment initiation. These findings advance our understanding of therapeutic response heterogeneity in this high-risk population and provide actionable insights for clinical decision-making.

HbA1c variability emerged as the strongest predictor of poor treatment response (adjusted OR 0.63 per 5% increase), surpassing traditional glycemic metrics like baseline HbA1c. This aligns with emerging evidence that glucose fluctuations induce greater oxidative stress and endothelial damage than chronic hyperglycemia alone (20). Our results extend recent findings by Mao et al., who demonstrated HbA1c variability predicts microvascular complications independent of mean HbA1c (21). Mechanistically, oscillating glucose levels exacerbate retinal inflammation through nuclear factor-kappa B (NF-κB) activation and VEGF upregulation (22), potentially diminishing anti-VEGF efficacy. NF-κB activation plays a pivotal role in the regulation of inflammation in retinal cells, where it induces the expression of various inflammatory cytokines, including IL-1β and TNF-α, that contribute to retinal damage and microvascular complications. Furthermore, the VEGF pathway is strongly influenced by NF-κB signaling, which upregulates VEGF expression, promoting angiogenesis and exacerbating retinal neovascularization, a hallmark of diabetic retinopathy. Recent research shows that inhibition of NF-κB can reduce VEGF levels and mitigate inflammatory responses, providing a therapeutic strategy for retinal diseases (23, 24). Additionally, NF-κB activation in retinal glial cells has been shown to enhance oxidative stress and cell migration, leading to further inflammation and vascular changes, which are critical in the progression of diabetic retinopathy (25). This biological plausibility supports our observation that each 5% increase in HbA1c CV reduced predicted response probability by 0.15 in the nomogram. These data underscore the importance of glycemic stability monitoring alongside conventional HbA1c targets in diabetic retinopathy management.

The significant association between FA non-perfusion area and treatment resistance (aOR 0.68 per 10% increase) highlights retinal ischemia as a key therapeutic determinant. Our findings corroborate optical coherence tomography angiography (OCTA) studies by Xu et al., who identified capillary nonperfusion as a predictor of anti-VEGF requirement in diabetic retinopathy (26). However, our use of widefield fluorescein angiography (FA) provides superior peripheral ischemia assessment, particularly relevant in severe non-proliferative diabetic retinopathy where peripheral pathology predominates (27). In our cohort, the mean FA non-perfusion area was 37.4 ± 12.8%, with substantial variability reflecting the heterogeneous nature of retinal ischemia in diabetic eye disease. The clinical implications of this finding are substantial for treatment planning, as widefield FA assessment can be integrated into pre-treatment stratification protocols to identify patients with extensive non-perfusion who may benefit from treatment intensification beyond standard combination therapy (28). Our regression analysis shows that each 10% increase in non-perfusion area decreased response likelihood by approximately 12%, suggesting that patients with more extensive ischemia might benefit from either more frequent anti-VEGF dosing or earlier supplemental panretinal photocoagulation targeting ischemic quadrants. While we acknowledge inherent limitations in FA-based ischemia quantification including variable contrast timing affecting perfusion appearance, projection artifacts in areas of hemorrhage, and inter-grader variability (29), we implemented standardized acquisition protocols and semi-automated quantification with manual verification to enhance reliability. The pathophysiological basis likely involves ischemia-driven upregulation of non-vascular endothelial growth factor angiogenic factors (platelet-derived growth factor [PlGF], angiopoietin-2 [Ang-2]) that bypass VEGF blockade (30). This explains why patients with greater non-perfusion area had lower response rates in our cohort, suggesting such cases may benefit from early supplemental therapies like panretinal photocoagulation or next-generation dual-pathway inhibitors.

Baseline DRSS severity independently predicted diminished treatment efficacy (aOR 0.72 per grade increase), with ETDRS level 53 patients showing 40% lower response probability than level 43 counterparts. This observation challenges the assumption that combination therapy uniformly benefits all severe NPDR subtypes. Pathologically, higher DRSS grades reflect advanced neurovascular unit degeneration and capillary dropout, creating a microenvironment resistant to vascular stabilization (31). Our data align with Protocol W subgroup analyses where baseline DRSS modified aflibercept efficacy in NPDR (32), but we uniquely quantify this relationship for combination therapy. Importantly, the nomogram assigns differential weights to DRSS levels, enabling precise risk stratification unavailable in current binary classifications.

Serum albumin represents a novel systemic predictor of treatment response (aOR 1.85 per 0.5 g/dL increase), a finding with significant clinical implications. Hypoalbuminemia reflects chronic inflammation and endothelial dysfunction, both implicated in diabetic retinopathy progression (33). Mechanistically, albumin maintains endothelial integrity by scavenging reactive oxygen species and inhibiting VEGF expression (34). Our study demonstrates its predictive value specifically in NPDR combination therapy. The 17% absolute probability gain per 0.5 g/dL increase suggests nutritional optimization and inflammation control may synergize with ocular treatments—an easily modifiable factor overlooked in current guidelines.

Our nomogram’s performance (AUC >0.75 in both cohorts) compares favorably with existing diabetic retinopathy prediction tools. The DRCR.net Protocol T model for anti-VEGF monotherapy in DME achieved AUC 0.68 (35), while machine learning algorithms using OCT biomarkers reached AUC 0.79-0.85 in research settings (36). The practical advantage of our model lies in its clinically accessible predictors, avoiding specialized imaging or genetic testing required by alternatives like other model (37). The robust validation performance (calibration slope 0.95, Hosmer-Lemeshow P=0.661) further supports real-world applicability. Importantly, decision curve analysis confirms clinical utility across therapeutically relevant thresholds, with net benefit peaking at 20% probability where clinical uncertainty is greatest.

The nomogram enables several practice-changing applications. First, it identifies high-probability responders (>75%) who may require less intensive monitoring. Second, it flags low-probability responders (<40%) who might benefit from treatment augmentation—such as earlier transition to faricimab (VEGF-angiopoietin-2 [Ang]2 inhibitor) in high-ischemia cases (38), or continuous glucose monitoring (CGM)-guided glycemic control for high-variability patients (39). Third, it provides objective criteria for clinical trial enrichment, potentially accelerating therapeutic development. The point-based scoring system facilitates rapid bedside calculation, addressing the implementation gap seen in complex machine learning models.

Several limitations warrant consideration. First, the retrospective design introduces potential selection bias, though temporal validation mitigates this concern. Second, FA analysis had 15% missing data despite standardized protocols. Future iterations could incorporate optical coherence tomography angiography (OCTA)-based nonperfusion metrics that correlate strongly with FA (26). Third, the model doesn’t account for specific laser parameters, though our analysis found no significant association. Fifth, our assessment of HbA1c variability was constrained by the retrospective nature of the data, with potential inconsistencies in the number and timing of available measurements. While we required a minimum of four measurements over the previous year for CV calculation, the irregular intervals between measurements may have affected our estimation of glycemic variability. Future prospective studies with standardized, equally-spaced HbA1c sampling protocols would strengthen the reliability of this predictor. Finally, we didn’t analyze molecular biomarkers like inflammatory cytokines that might enhance precision.

## 5 Conclusion

This study establishes a validated nomogram integrating glycemic variability, retinal ischemia, disease severity, and systemic nutrition to predict anti-VEGF-laser combination therapy response in severe NPDR. The model’s robust performance and clinical accessibility address a critical need for personalized treatment strategies in this sight-threatening condition. Future work should focus on prospective validation, integration with OCTA biomarkers, and assessment of nomogram-guided treatment algorithms.

## Data Availability

The original contributions presented in the study are included in the article/Supplementary Material. Further inquiries can be directed to the corresponding author.
